# Acute kidney injury after colorectal surgery with prophylactic ureteral stents

**DOI:** 10.1007/s00464-024-10941-5

**Published:** 2024-06-11

**Authors:** Assar Rather, Adrianne Fisher, Kelly Gardner, Nessreen Ghanem, Theodoris Katsichtis, Gary Siegelman, John D. Mannion

**Affiliations:** Bayhealth Medical Center, 640 South State Street, Dover, 19901 DE USA

**Keywords:** Acute kidney injury, Ureteral stent, Complications

## Abstract

**Background:**

After colorectal surgery, acute kidney injury (AKI) results from a complex interplay of multiple independent causes and preventive measures that occur during the hospitalization. Prophylactic stenting for ureter identification has been identified as a potential cause, but the evidence is conflicting, possibly because of differing baseline characteristics and procedure-related approaches.

**Objective:**

This retrospective cohort study assesses the role of stents in the etiology of AKI after determining the independent predictors of AKI.

**Methods:**

From a population of 1224 consecutive colorectal patients (from 8/1/2016 through 12/31/2021), 382 (31.2%) received ureteral stents, and propensity score matching was used to create stented and control groups. Emergent cases and patients with sepsis were excluded from the analysis. Previously identified independent predictors of AKI, minimally invasive procedures, and a history of diabetes mellitus were used as criteria to create two balanced groups.

**Results:**

Baseline demographic characteristics and procedure-related factors baseline factors were similar between the groups. There was no difference in the rate of AKI between stented patients and controls (*P* = 0.82), nor was there any difference in postoperative complications, such as chronic renal insufficiency (CRI, *P* = 0.49), average postoperative creatinine (*P* = 0.67), urinary tract infections (UTI, *P* = 0.82), any postoperative infection (*P* = 0.48), in-hospital complications (*P* = 1.00), length of stay (LOS, *P* = 0.15), and 30-day readmissions (*P* = 0.79).

**Conclusions:**

In a population of patients where stenting was frequently employed, ureter stents placed for identification did not appear to cause AKI or AKI-related complications.

**Graphical Abstract:**

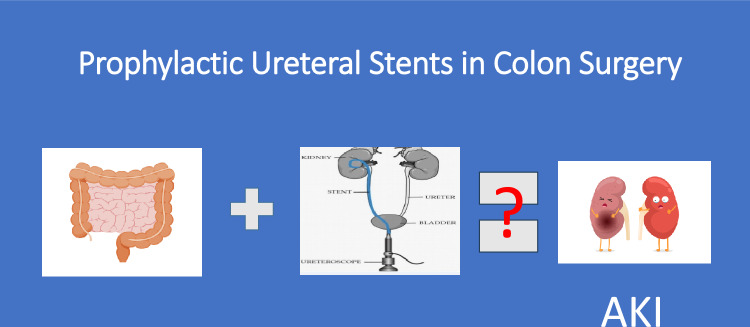

The benefit of the common use of prophylactic ureteral stenting for ureteral identification before colorectal surgery is questionable [1–4]. The incidence of direct ureteral injury from stent placement is low, but worrisome complications have been documented [5–7]. Further, the effectiveness of stents in avoiding surgical injury has been suggested [8] but has not yet been proven [9] when only open procedures are included [10] and with minimally invasive surgery [11]. Nonetheless, there continue to be reasons why many surgeons see a benefit in stents: complications can be severe if a ureteral injury is identified in the postoperative period, often requiring reoperations during a vulnerable time or prolonged nephrostomy drainage, and are associated with sepsis [12]. A ureteral injury identified at surgery may be much easier to remediate, and a protracted postoperative course is avoided.

Prophylactic stenting might be even more encouraged if there were minimal side effects. The most reported complication of stents is acute kidney injury (AKI), but the contribution of stents to AKI is uncertain. Several studies have noted a significant increase in AKI with stenting [13, 14], but another study found no increase [15]. Confounding by indication is one problem that can make the data difficult to interpret: high-risk patients are most likely to benefit from prophylactic stent placement, but these high-risk patients are also most likely to experience postoperative AKI that is unrelated to stenting. A stent is only one of multiple factors that can cause or prevent AKI, and different institutions may have a different proportion of these factors.

We recently analyzed the potential role of inflammation as a cause of AKI after colorectal surgery [16]. We noted that the causes of AKI are multiple and varied, but that when analyzing many predictors, stenting was not a univariate predictor of AKI. In this report, we further explore the role of stents and AKI with propensity score matching to compare non-stented with stented patients, with multiple independent predictors of AKI used to create two matched groups. Short of a randomized trial, comparing two similar groups might give more information on whether stents contribute to postoperative kidney injury.

## Materials and methods

Independent predictors of AKI were examined in 1224 consecutive patients undergoing colorectal surgery or stoma procedures at Bayhealth Medical Center from August 2016 through December 2022 [16]. The potential contribution of peri-surgical inflammation to the development of AKI and other risk factors was investigated in this retrospective cohort study using Strobe 2021 standards. AKI was defined with the Kidney Disease Improving Global Outcomes (KDIGO) criteria. Using stents for ureter identification was not predictive of AKI on logistic regression, but the potential for confounding led to a propensity score analysis reported here.

Routine demographic data were extracted from Epic. A list of patients with congestive heart failure (CHF) or malignancy was built from a CHF registry and a Problem List from Epic.

**Fig. 1:**
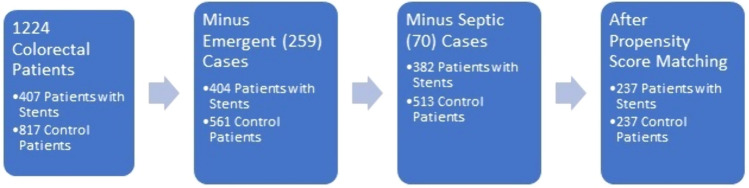
Flowchart for selection of patients for propensity matching

Patients with emergent operations or active infections diagnosed at the time of surgery were not included in this analysis. Six of the 7 Independent predictors of AKI previously determined were used to balance stented and unstented patients for this study. Male sex, age ≥ 60, vancomycin use, average hemoglobin over three days, procedure duration, and transfusion volume were equalized between the groups. The WBC on the first postoperative day was not used to discriminate between patients since an elevated WBC could be considered an outcome of a traumatic ureteral insertion. For this study, procedure duration was modified by subtracting 45 min from the anesthesia time in patients with stents to equalize the actual operation time between the two groups. Finally, a history of diabetes mellitus and the use of minimally invasive approaches, predominantly laparoscopic, were included to enhance the evenness between groups further. After propensity matching, there were 237 patients with stents and 237 controls. There was no significant difference between the groups for any of the predictors of AKI that were used to balance the groups. Figure 1 is a flow diagram of the study group.

Ureteral stents were generally placed for left-sided colon resections, abdominal perineal resections, and right-sided colon resections in patients with complicated Crohn's disease. Urologic surgeons performed the stenting procedure at the request of colorectal surgery. Typically, after cystourethroscopy, each ureteral orifice was cannulated with a Sensor wire (Boston Scientific, Marlborough, MA), and either an open-ended ureteral stent (6-French, Stryker) or a non-lighted stent (5-French, COOK) was advanced to the proximal ureter. Light-emitting fibers were inserted into the stents. Fluoroscopy was used infrequently. The stents were uncapped, with urine flowing into the bladder during the operation. The stents were removed at the end of the surgery.

As in the previous study, any patient with an increase of creatinine of ≥ 0.3 mg/dl during the first 48 h after surgery or an increase of 1.5 times the preoperative creatinine from day three through day seven was diagnosed with AKI. Additional perioperative complications were also assessed. Urine cultures and postoperative infections were considered positive with the identification of pathogenic bacteria, not normal flora. In-hospital complications were extracted from medical records using Conduent-Midas Health Analytics Solutions (Florham Park, NJ). Respiratory and cardiopulmonary failure and arrest, deep vein thrombosis, myocardial infarction, and stroke were included. 30-day readmissions and length of stay (LOS) were also calculated.

This analysis of colorectal patients was approved by the Bayhealth Institutional Review Board.

Continuous data were expressed as means ± standard deviations; categorical data were expressed as percentages. Unpaired t-tests and tests for two proportions were used to test for differences between groups. Propensity score matching was performed with software from XLSTAT (Lumivero (2023) XLSTAT Statistical and Data Analysis Solution. New York, USA.) The corresponding author performed a statistical analysis.

## Results

Two hundred thirty-seven patients with stents were matched with 237 unstented controls. The matching resulted in balanced groups for all the variables chosen, as seen in Table 1 for demographic factors, except for a higher percentage of Caucasian patients and a lower percentage of patients with malignancy in the stented group. Procedure-related factors, seen in Table 2, were similar.

**Table 1:** Demographic factors

	No Stents	Stents	*P* value
Patients	237	237	
Male	120 (50.6%)	111 (46.8%)	0.462
Age ≥ 60	160 (67.5%)	161 (67.9%)	1
Diabetes	52 (21.9%)	48 (20.3%)	0.736
CHF	16 (6.8%)	13 (5.5%)	0.701
Caucasian	172 (72.6%)	199 (84.0%)	0.003
Malignancy	92 (38.8%)	64 (27.0%)	0.008

**Table 2:** Procedure-related factors

	Patients	Laparoscopic surgery	Average Hg (g/dl)	Duration (minutes)	Transfusion volume (ml)	Vancomycin
No stents	237	151 (63.7%)	11.45 ± 1.76	263.5 ± 84.6	95.0 ± 303.0	3 (1.3%)
Stents	237	139 (58.6%)	11.58 ± 1.70	266.1 ± 76.0	78.7 ± 346.1	3 (1.3%)
*p* value		0.299	0.417	0.720	0.586	1.000

Laparoscopic surgery includes a few cases of robot or robot-assisted surgery; Hg average is from preoperative and first 3 postoperative days

Fluoroscopy was required in 36 (15.2%) of patients. Laterality occurred as follows: bilateral stents were placed in 212 (89.5%) patients, unilateral stents in 16 (6.8%) patients, unknown in 3 (1.3%) patients, and coding issues were noted in 5 (2.1%). Lighted stents were placed 84.0% of the time (68.4% bilateral lighted and 15.6% mixed (one lighted, one non-lighted). Non-lighted stents were used solely in 12.2% of patients and unknown in 2.8%.

Stent insertions were generally routine and uncomplicated, but on occasion, modification of the insertion technique was required. Unexpected findings included preoperative ureteral obstruction or hydronephrosis, difficulty with ureteral cannulation, or anatomical variations.

Table 3 shows the preoperative and postoperative creatinine values, the percentage of patients who developed AKI, and measures of long-term renal function. There was no difference in the average preoperative or postoperative creatinine values. There was no difference in the rate of acute kidney injury between the groups (*P* = 0.82). There was no difference in the average creatinine drawn between 3 months and one year after surgery. Finally, there was no difference in the percentage of patients with either a sensitive (≥ 0.3 + preoperative creatinine) or strict (≥ 1.5 * preoperative creatinine) definition of chronic renal insufficiency (CRI) after surgery. In summary, stents had no apparent effect on postoperative renal function.

**Table 3:** Renal complications in stented and unstented patients

	Patients	Preoperative Cr (mg/dl)	Postoperative Cr (mg/dl)	AKI	Long-term Creatinine (mg/dl)	CRI (sensitive)	CRI (strict)
No stents	237	0.97 ± 0.40	0.90 ± 0.45	48 (20.3%)	1.06 ± 0.62	21 (8.9%)	17 (7.2%)
Stents	237	0.94 ± 0.39	0.92 ± 0.52	51 (21.5%)	1.01 ± 0.47	16 (6.8%)	9 (3.8%)
*P* value		0.47	0.67	0.82	0.35	0.49	0.16

Long-term creatinine sampled between 3 months and 1 year after surgery; CRI sensitive: chronic renal insufficiency, where long-term creatinine was ≥ 0.3 + preoperative creatinine; CRI strict: chronic renal insufficiency, where long-term creatinine was ≥ 1.5 * preoperative creatinine

There was no difference between the groups in the incidence of postoperative complications, as seen in Table 4. The ureteral stents were associated with gross postoperative hematuria in a patient treated with bladder irrigation. There were no returns to the operating room for a stent complication, evidence of perforation, or bleeding requiring intervention.

**Table 4:** Hospital complications in stented and unstented patients

	Patients	Positive urine culture	Positive any culture	30-Day readmission	Non-infectious complications	In-hospital complications	LOS (Median, IQR)
No stent	237	9 (3.8%)	25 (10.5%)	32 (13.5%)	10 (4.2%)	33 (13.9%)	5.0 (IQR 4.0–8.0)
Stent	237	11 (4.6%)	31 (13.1%)	35 (14.8%)	4 (1.7%)	34 (14.3%)	6.0 (IQR 4.0–10.0)
*P* value		0.82	0.48	0.79	0.17	1.00	0.15

Non-infectious complications include respiratory or cardiopulmonary failure, DVT/PE, acute myocardial infarction or stroke, or cardiac or pulmonary arrest; In-hospital complications include patients with a positive pathogenic culture or non-infectious complication(s). A patient with 1 or more in-hospital complications was considered as a complication. LOS in days(median and interquartile range)

No ureteral injuries occurred during the colorectal surgery.

## Discussion

Acute kidney injury after colorectal surgery is not inconsequential [17]. An increased rate of perioperative infections [18, 19], a persistence of kidney dysfunction for extended periods in a sizable percentage of patients [20], increased rates of serious CV-related complications, and a small but detectable mortality risk can accompany even benign-appearing increases in perioperative creatinine [21–23]. These complications remained hidden by their small magnitude but were statistically and clinically meaningful. It is appropriate to examine AKI after ureteral stenting to make sure that prophylactic stent placement is not associated with poor outcomes.

Several factors make the evaluation of AKI after stenting for colorectal surgery difficult. First, the background incidence of AKI is high, which might make small increments challenging to identify. Second, there are multiple predictors of AKI, most of which cause AKI, but some might prevent or alleviate it. Ideally, all these clinical factors, which can vary among surgeons or institutions, should be examined to assess their independence. Third, in most studies, ureteral stents are placed in only a small percentage of patients, usually with the highest anatomic or physiologic risk. A higher incidence of AKI might reflect a higher risk. Even if many risks are accounted for, the fact that a urinary stent is placed is an intuitive risk assessment by surgeons, which is difficult to quantify.

Three previous studies have examined the effect of ureteral stents on AKI using the KDIGO criteria. Hassinger et al. noted an 11.5% incidence of AKI in the overall population, using a strict KDIGO criterion (creatinine increase ≥ 1.5 the baseline value), and reported a 32.6% incidence in stented patients. Using the same KDIGO criteria, Matkov et al. noted a lower overall incidence of AKI (0.8%) but also a significant increase associated with stenting (5.8%) [14]. The difference in these studies in the overall incidence of AKI might be related to a selective versus liberal indication for stent placement. Still, they both highlighted a potential concern with stent use. Even minor increases in creatinine, lower than the KDIGO definition of AKI, are associated with increases in mortality [21]. In contrast, Schmeid et al., in a study examining many predictors with liberal use of stents, noted an incidence of AKI of 15.9% using a strict criterion in unstented patients but no significant increase with ureteral stents [15]. However, the authors did note that lighted stents were associated with an increase in AKI when using the more sensitive criteria (Cr increase of ≥ 0.3 mg/dl within the first 48 h of surgery). The different conclusions concerning AKI and stent use may be a result of varying indications for stent employment and distinct populations of patients with differing combinations of factors that either promote or prevent AKI.

In this study, where patients were matched with risk factors associated with AKI, there was no increase in AKI or even a slight increase in perioperative creatinine with ureteral stenting, even though lighted stents were used predominantly and assessed with sensitive KDIGO criteria. Further, there was no increase in CRI or creatinine when kidney function was evaluated between 3 months and one year after surgery. There was also no evident increase in urinary tract infections, any postoperative infections, or other postoperative complications associated with AKI.

## Limitations

Surgical risks are difficult to quantify and may not be adequately accounted for when balancing the characteristics of the non-stented and stented groups.

## Conclusion

The placement of prophylactic ureteral stents before colorectal surgery to help avoid ureteral injury or make an inadvertent injury easier to identify requires a careful balancing of risks and benefits. There is evidence that stenting can help prevent surgical ureteral damage [8], but the benefits of stenting are far from certain. Stents have downsides: procedure time is significantly prolonged, and ureteral perforation and temporary obstruction can occur. The absence of either AKI or AKI-related postoperative complications from stenting, evident here, combined with a satisfactory experience with stent insertion, leads us to continue to favor prophylactic ureteral stenting after a careful risk assessment.
